# Helical Multi-walled Carbon Nanotubes as an Efficient Material for the Dispersive Solid-Phase Extraction of Low and High Molecular Weight Polycyclic Aromatic Hydrocarbons from Water Samples: Theoretical Study

**DOI:** 10.1007/s11270-018-3884-0

**Published:** 2018-07-14

**Authors:** Monika Paszkiewicz, Celina Sikorska, Danuta Leszczyńska, Piotr Stepnowski

**Affiliations:** 1 Department of Environmental Analysis, Faculty of Chemistry, University of Gdansk, Wita Stwosza 63, 80-308 Gdansk, Poland; 2 Department of Civil and Environmental Engineering, Interdisciplinary Nanotoxicity Center, Jackson State University, 1400 John R. Lynch Street, Jackson, MS 39217 USA; 3 Laboratory of Molecular Modeling, Department of Theoretical Chemistry, Faculty of Chemistry, University of Gdansk, Wita Stwosza 63, 80-308 Gdansk, Poland

**Keywords:** Multi-walled carbon nanotubes (MWCNTs), Adsorption, Dispersive solid-phase extraction (dSPE), Principle component analysis (PCA), Polycyclic aromatic hydrocarbons (PAHs), Molecular modeling, Analytical chemistry, Polarizable continuum model (PCM)

## Abstract

The differences in effectiveness of multi-walled carbon nanotubes (MWCNTs) as the dispersive solid-phase extraction (dSPE) sorbent for the selective extraction of polycyclic aromatic hydrocarbons (PAHs) were explained on the basis of theoretical study. It was observed that for low molecular weight PAHs, the recoveries using non-helical and helical MWCNTs were similar. In contrary, for PAHs containing five or more aromatic rings, the extraction efficiency was higher using HMWCNTs than for non-helical ones. Principle component analysis (PCA) as well as providing structural parameters and interaction energies for adsorption processes (PAH + CNT → PAH-CNT) have been used for this purpose. All the PAH + CNT → PAH-CNT adsorption processes considered were found to be thermodynamically favorable. However, the adsorption energies (*E*_ads_) for PAHs and the helical carbon nanotube surface estimated for the B(a)P-HCNT and I(1,2,3-cd)P-HCNT are substantially less negative than those observed for PAH molecules interacting with the non-helical CNT. Namely, the *E*_ads_ calculated in simulated aqueous environment for the B(a)P-MWCNT(6,2) and I(1,2,3-cd)P-MWCNT(6,2) were respectively − 43.32 and − 59.98 kcal/mol, while values of only − 7.75 kcal/mol (B(a)P-HCNT) and − 9.13 kcal/mol (I(1,2,3-cd)P-HCNT) were found for the corresponding PAH-HCNT systems. Therefore, we conclude that the replacement of MWCNTs with HCNTs leads to PAH-HCNT systems in which the interaction energies are much smaller than those estimated for the corresponding PAH-MWCNT systems. HMWCNTs are therefore recommended as the dSPE sorbent phase for the extraction of both low and high molecular weight PAHs from water samples.

**Electronic supplementary material:**

The online version of this article (10.1007/s11270-018-3884-0) contains supplementary material, which is available to authorized users.

## Introduction

The use of carbon nanotubes (CNTs) as sorbents in extraction techniques is one of the most discussed topics in analytical chemistry (Ravelo-Pérez et al. 2010; Pyrzynska 2011, 2013; Scida et al. 2011; Paszkiewicz et al. 2016; Jakubus et al. 2017). The popularity of these materials is associated with their tunable and unique properties. Several types of CNTs are relatively inexpensive materials with a wide range of lengths, diameters, and number of walls, which can be modified by functional groups in order to improve their properties in specific applications (Popov 2004; Wepasnick et al. 2010; Masotti and Caporali 2013; Trakakis et al. 2013; Fazelirad et al. 2014, 2015; Hu et al. 2015). The high surface area of CNTs allows their use in a much smaller amount as compared to classic adsorbents, and the thermal stability of CNTs allows their easy regeneration and reuse. Recently published works have shown that, in general, CNTs can be used as the stationary phase in SPE, SPME, and SBSE for the isolation of organic or inorganic analytes. Multi-walled carbon nanotubes (MWCNTs) have been used for the enrichment of phenolic compounds, phthalate esters, pesticides, drugs, and others (Cai et al. 2003; Fang et al. 2006; Niu et al. 2007; Suárez et al. 2007a, b; Zhao et al. 2007; El-Sheikh et al. 2007, 2008; Ravelo-Pérez et al. 2008; Al-Degs et al. 2009; Guan et al. 2010; Gethard and Mitra 2011; Abdolmohammad-Zadeh and Sadeghi 2012; Bhadra et al. 2012; Sun et al. 2014; Hou et al. 2014). As for the extraction of PAHs, CNTs have been used as sorbents only in several papers (Wang et al. 2007; Ma et al. 2010; Wu et al. 2010; Menezes et al. 2015). In all, the results have shown that the extraction efficiency was comparable or higher than with using conventional stationary phases. Wang et al. (2007) developed a simple method to determine PAHs in tap and river waters using MWCNTs of 30–50 nm (OD) in combination with HPLC-UV. Multi-walled carbon nanotubes were also used as the adsorbent for the determination of 16 polycyclic aromatic hydrocarbons in environmental water samples (Ma et al. 2010). MWCNTs (150 mg) presented a higher extraction efficiency of the 16 PAHs than the commercial C18 column. A flow injection solid-phase extraction system using a micro-column packed with MWCNTs for the determination of PAHs was also described (Wu et al. 2010). The best results were obtained when methanol was used as the elution solvent and the carbon nanotubes had an outer diameter of 60–100 nm. Another method of sample preparation and the enrichment of PAHs from environmental matrices using hybrid magnetic carbon nanotubes (mCNTs) has been proposed by Menezes et al. (2015). The hybrid mCNTs contain hydrophilic and hydrophobic parts which allow the maintenance of a stable dispersion of nanoparticles in water and the effective adsorption of PAHs. The authors found that the mCNTs are more effective sorbents than polydimethylsiloxane (PDMS) for all PAHs of molecular weights lower than chrysene. This is consistent with the results obtained in our previous work (Paszkiewicz et al. 2017). While selecting the conditions for simultaneous extraction of PAHs and metal ions, it was observed that recoveries of high molecular weight PAHs was below 70% using unmodified and modified MWCNTs. This is probably connected with the very strong adsorption on the surface of some CNTs, which makes the desorption step very difficult, while using helical MWCNTs, the extraction yield of low and high molecular weight PAHs was significantly higher. Therefore, to further understand the adsorbing characters of PAHs onto carbon nanotubes, we carried out a theoretical investigation on the interaction mechanisms between PAH molecules and MWCNTs using the PM6 method. The conclusion is supported by employing chemometric analysis as well as providing structural parameters and interaction energies for adsorption processes (PAH + CNT → PAH-CNT).

## Materials and Methods

### Chemicals

The PAH standard solution including phenanthrene (Phen), anthracene (Ant), fluoranthene (Fluo), pyrene (Pyr), chrysene (Chr), benzo[a]anthracene (B[a]A), Perylene (Per), benzo[a]pyrene (B[a]P), indeno[1,2,3-cd]pyrene (I[1,2,3-cd]P), and benzo[k]fluoranthene (B[k]F) was purchased from Sigma-Aldrich. The PAH stock solution was prepared in an acetone:dichloromethane (2:1, *v*/*v*) mixture in a concentration of each compound at 2 μg mL^−1^ and kept at 4 °C in darkness. The PAH working solutions were prepared by the proper dilution of the stock solution. Dichloromethane (DCM), methanol (MeOH), acetonitrile (ACN), and acetone (ACT) were obtained from POCH (Gliwice, Poland). The deionized water used in all experiments was purified by Hydrolab System (Gdańsk, Poland).

### Carbon Nanotubes

Three different types of carbon nanotubes, helical multi-walled carbon nanotubes 100–200 nm OD, length 1–10 μm (HMWCNTs); multi-walled carbon nanotubes > 50 nm OD, length 10–20 μm (50MWCNTs); and multi-walled carbon nanotubes < 8 nm OD, length 10–50 μm (8MWCNTs), were purchased from Cheap Tubes Inc. (Cambridgeport, USA). MWCNTs and HMWCNTs were characterized in order to determine its surface area and morphology using Brunauer–Emmett–Teller (Micromeritics ASAP® 2420, nitrogen as adsorbed gas) and high-resolution scanning electron microscope Quanta 3D FEG (SEM/FIB, scanning electron microscope/focused ion beam)).

### Gas Chromatography-Mass Spectrometry

An Agilent gas chromatograph coupled to a mass QP 2010 SE spectrometer (Shimadzu, Maryland City, MD, USA) was used. The mass spectrometer was used in EI mode (ionization energy of 70 eV) and set to scan a mass range from 45 to 700 amu. The samples were introduced through the gas chromatograph, equipped with a 30-m × 0.25-mm I.D., an Optima-5 silica capillary column (Restek), and a 0.25-μm thick film. The oven temperature was increased from 60 to 300 °C at 4 °C/min. The injector temperature was 300 °C and the carrier gas was helium. The injector was set to 280 °C, and the injections were done using the splitless mode.

### Sorption/Desorption Study

Two hundred and fifty milliliters of deionized water containing 0.5 μg L^−1^ of each PAH and 5% of *n*-propanol was transferred to a flask containing 100 mg of MWCNTs and shaken for 10, 20, 30, 45, 60, and 90 min. After that, the dispersed MWCNTs were passed through an empty SPE tube (Resprep, Bellefonte, USA) using a Baker SPE-12G™ vacuum manifold. After that, the CNTs were dried under a vacuum for 20 min. The analytes were eluted with the appropriate organic solvent (6 mL). The obtained extract was evaporated to dryness at 40 °C. The dry residue was re-dissolved in 100 μL of DCM, filtrated using 0.20-μm filters (Chromafils Xtra PET-20/25 from Macherey-Nagel), and analyzed by GC-MS.

In the next step, an internal standard solution (9-methylanthracene) was added to the water phases to a final concentration of 0.5 μg L^−1^ and a triplicate extraction using 50 ml of DCM was done. The obtained extracts were combined, evaporated to dryness using a rotary evaporator, and re-dissolved in 1 mL of DCM. The concentrations of PAHs in DCM extracts were determined by GC-MS. The following formula was used for the adsorption rate assessment (Eq. 1):

1 $$ A\ \left(\%\right)=100-\left(\mathrm{C}1/\mathrm{C}0\right)\times 100 $$

where *A* indicates the adsorption rate, C0 is the initial concentration of the PAH in the water sample, and C1 refers to the concentration of the PAH in the water sample after MWCNT adsorption, calculated using the internal standard method.

### PCA

In order to explore the structural similarity of the studied PAHs, we performed principal component analysis (PCA, supporting Section 2.1). This approach is based on the axiom that some of the features (in our case, molecular descriptors) that describe samples/cases (in our case, PAHs) are correlated with each other, and according to this fact, those features carry the same information about the samples. In other words, the PCA approach was adopted for grouping the studied PAHs based on their structural similarity. Since the usefulness of this technique for describing the properties of different chemicals was earlier confirmed (Sikorska and Puzyn 2015; Mioduszewska et al. 2017), and given the enormity of advantages resulting from the application of this type of approach (such as speed, low cost, and safety), this approach was performed in the present contribution. In particular, we have presented the structures of PAHs in the space of the first and second principle components (score plot), in accordance with the demonstrative criterion (Abdi and Williams 2010). We assumed that the objects (PAHs) located close to each other on the plot were structurally similar. A physical interpretation was assigned to each PC based on the Malinowski rule (only the contributions of descriptors with normalized loadings higher than 0.7 were significant) (Praus 2005).

### Quantum Calculations

The adsorption processes (in which the carbon nanotubes react with various PAH molecules) were investigated by means of the semi-empirical (PM6) method to obtain the stationary point structures (i.e., minimum energy). The Hessian matrix and subsequently the normal modes for all stationary points were calculated to confirm the optimized structures as the local minima on the potential energy surfaces. For the reaction path PAH-CNT → PAH + CNT, the electronic energies obtained from PM6 were corrected by the thermal and zero-point energy contributions to obtain the enthalpies (Δ*H*_r_^298^) and entropies (Δ*S*_r_^298^) of the processes. The Gibbs free energies (Δ*G*_r_^298^) of the decomposition processes of the PAH-CNT systems considered were calculated for the temperature T of 298.15 K and for the pressure p of 1013 hPa (1 atm).

To approximate the effect of the surrounding solvent molecules on the adsorption energies, we employed the polarized continuum (PCM) solvation model (Miertuš et al. 1981; Miertus̃ and Tomasi 1982) within a self-consistent reaction field treatment, as implemented in the Gaussian 09 program. The PCM calculations in aqueous solution were carried out with the same level of theory (i.e., PM6) as that for the isolated species.

All calculations were performed with the Gaussian09 package (Frisch et al. 2009). Carbon nanotube structures were constructed using Nanotube Modeler software (Nanotube Modeler 2015).

## Results and Discussion

### Types of Carbon Nanotubes

**Fig. 1:**
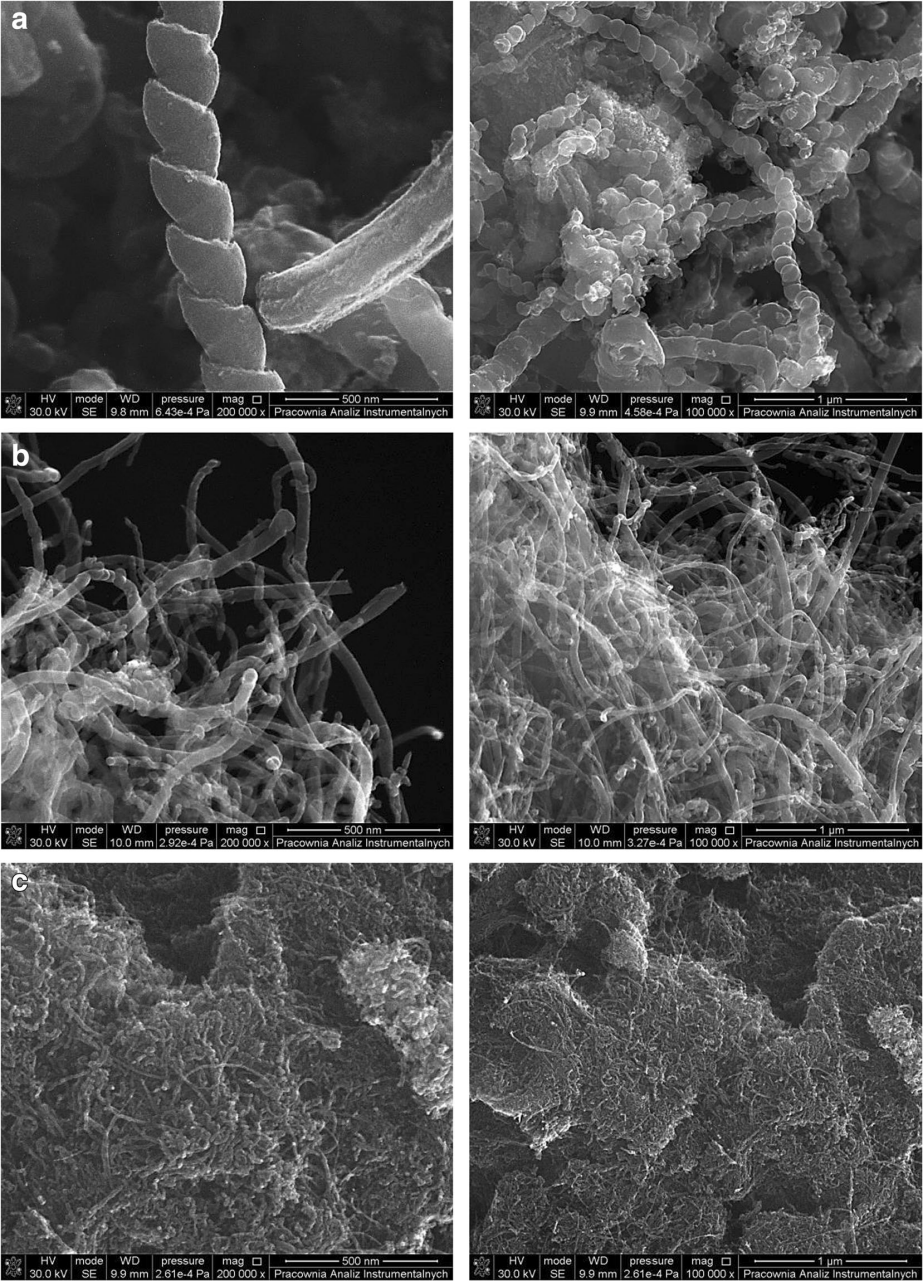
Comparison of **a** helical MWCNTs, **b** 50MWCNTs, and **c** 8MWCNTs by HR-SEM

**Fig. 2:**
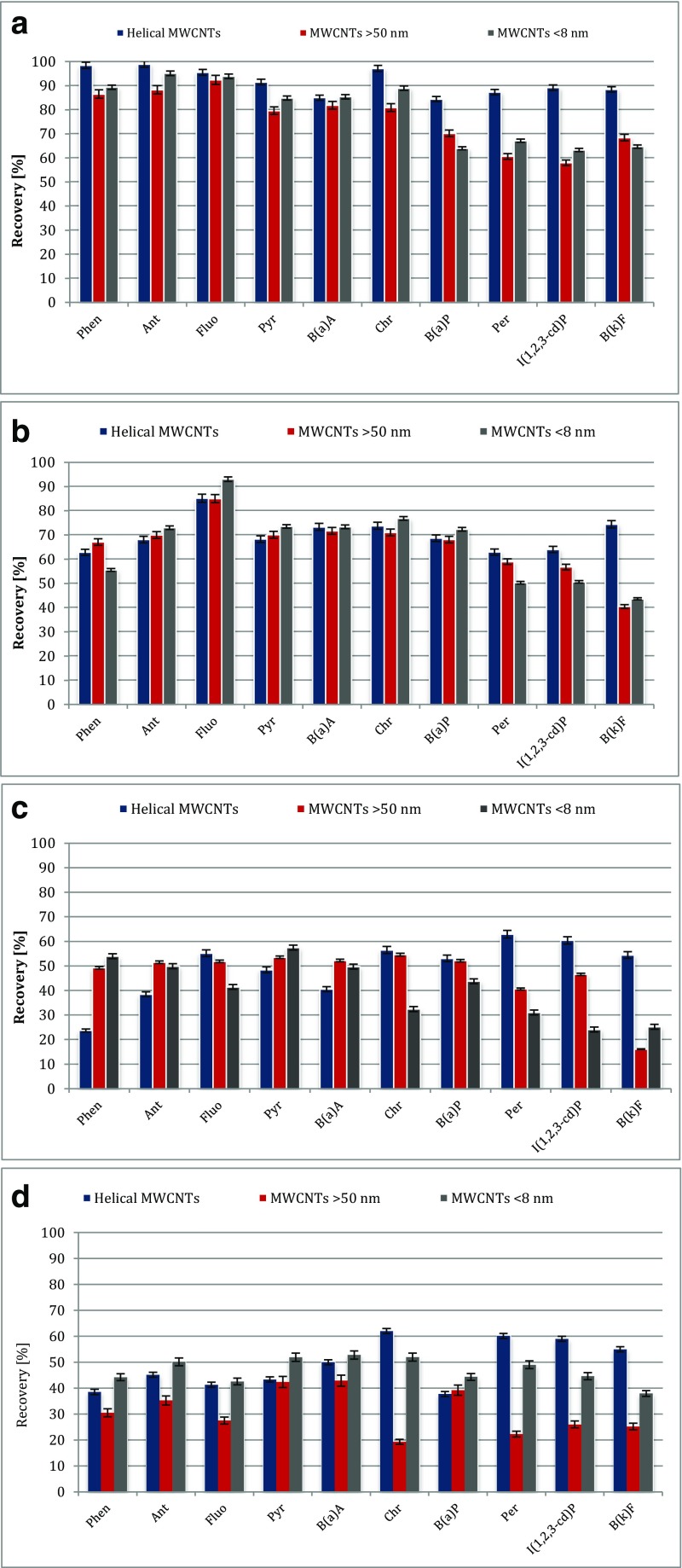
Effect of the eluent type on the recovery of PAHs **a** dichloromethane, **b** *n*-hexane, **c** dichloromethane/acetone mixture, **d** acetonitrile. (Each PAH concentration 0.5 μg L^−1^; packing material 25 mg of CNT, sample volume 250 mL)

The structure and electronic properties of carbon nanotubes (CNTs) allow the interactions with organic molecules by non-covalent forces such as hydrogen bonding, π-π stacking, electrostatic forces, van der Waals, and hydrophobic interactions. Two MWCNTs, i.e., 8MWCNT (BET-specific surface area 437.5 ± 1.5 m^2^ g^−1^) and 50MWCNT (BET-specific surface area 86.0 ± 0.6 m^2^ g^−1^), and helical MWCNTs (HMWCNT; BET-specific surface area 103.3 ± 0.4 m^2^ g^−1^) were chosen as sorbents to test their capability for the sorption of PAHs. Comparison of MWCNTs and helical MWCNTs morphology by HR-SEM is shown in Fig. 1. Because the CNTs have a high sorption ability for PAHs (Table 1), the composition of the eluent was optimized for both MWCNTs and for HMWCNTs. Consequently, four elution solvents, acetonitrile, dichloromethane, *n*-hexane, and a mixture of dichloromethane and acetone, were tested. From Fig. 2a, it is shown that the best results were obtained for HMWCNTs and dichloromethane as the eluent with the recovery value from 84.0 to 98.9%, while for 8MWCNTs and 50MWCNTs, recoveries were lower and read 63.3–95.1 and 58.0–92.4%, respectively. Similar dependencies were observed in the case of *n*-hexane and acetonitrile as the elution solvent; however, the extraction efficiency values were significantly lower. The remaining tested solvents have an insufficient elution strength, and the extraction efficiency was significantly lower. Explicitly, using *n*-hexane as a solvent provides the extraction recoveries of 10 PAHs in the range of 62.9–85.2% using HMWCNTs, 40.5–84.9% using 50MWCNTs, and 43.6–93.1% for 8MWCNTs. For the mixture of dichloromethane/acetone, the recoveries ranged from 16.0 to 62.9% and were similar for all the tested types of CNT. For the acetonitrile, the recoveries ranged from 19.3 to 62.0% and were similar for all tested types of CNT. Having discussed the solvent elution issue, we move on to the analysis of the influence of PAHs and CNTs on the extraction efficiency. According to our results, recoveries of low molecular weight PAHs were dependent neither on the type nor outer diameter of the CNTs. Similar extraction efficiency values (ranging from 81 to 99%) were obtained using all tested CNTs. The highest recoveries in the range of 91 to 99% were obtained using HMWCNTs, while the recoveries for both tested MWCNTs were only slightly lower (within the range of 80–95%). In particular, the recovery values for phenanthrene were as follows: 98.4% for HMWCNTs, 86.5% for 50MWCNTs, and 89.4% for 8MWCNTs. The same trend was observed for the other low molecular PAHs, for example, the recoveries of pyrene were 91.5% for HMWCNTs, 79.6% for 50MWCNTs, and 84.9% for 8MWCNTs. However, for higher molecular weight analytes (with five and more aromatic rings), the extraction efficiency was significantly lower when 50MWCNTs and 8MWCNTs were used instead of the application of HMWCNTs. Explicitly, for perylene, the extraction efficiency was 87.3% using HMWCNTs, while for 50MWCNTs and 8MWCNTs, it was only 60.6 and 67.2%, respectively. The same dependence was observed for benzo(k)fluoranthene, indeno(1,2,3-cd)pyrene, and benzo(a)pyrene (as depicted in Fig. 2a). Therefore, we conclude that low molecular PAHs combine with HMWCNTs to form a weakly bound PAH–HMWCNT complex. Such adducts are expected to be susceptible to fragmentation leading to a PAH recovery with a high extraction efficiency. Higher molecular weight PAHs form stronger bound PAH-MWCNT systems, which result in lower PAH recoveries. Our results are in agreement with previous studies and confirmed a very high extraction capability of CNTs for low molecular PAHs. The results obtained by Ma et al. (2010) indicate that the recoveries of low ring PAHs extracted by 150 mg MWCNTs were higher than those by C18. MWCNTs effectively adsorbed the PAHs (four to six rings) with recoveries ranging from 75.3 to 125.7%. The authors explain that PAHs are adsorbed onto MWCNTs by the coaction of a delocalized π bond interaction and physical adsorption. This interaction is much stronger than the hydrophobic interaction between PAHs and C18. The authors concluded that PAHs can be adsorbed more easily on MWCNTs than on C18; however, high molecular PAHs are not easily desorbed from CNTs. A high surface area (980 m^2^ g^−1^) microporous activated carbon was also used for the extraction of the PAH contaminants. Despite the high surface area, the activated carbon showed lower extraction efficiencies than the CNTs. This is likely related to the very strong adsorption in the microporous space which makes the desorption step very difficult. On the other hand, the usefulness of the three types of MWCNT with different outer diameters in flow injection solid-phase extraction for the adsorption of 16 PAHs was tested (Wu et al. 2010). The results show that the highest recovery values of 16 PAHs were obtained using MWCNTs with the largest outer diameter in the range of 60–100 nm. The authors presented that the recoveries for high molecular weight PAHs were below 60% using MWCNTs with an outer diameter in the range of 10–30 and 40–60 nm. Different approaches for sample preparation and the enrichment of PAHs from environmental matrices have been proposed by Menezes et al. (2015). The authors have used hybrid magnetic carbon nanotubes as a sorbent for the isolation of PAHs. These N-doped amphiphilic CNTs contain hydrophilic and hydrophobic parts which allow the maintenance of a stable dispersion of nanoparticles in water and the effective adsorption of PAHs. After adsorption, the CNTs can easily be removed from the medium by a simple magnetic separation. The authors found that the mCNTs are a more effective sorbent than polydimethylsiloxane (PDMS), or activated carbon, with a recovery ranging from 80.50 ± 10 to 105.40 ± 12%. However, the developed method was applied for the isolation of low molecular weight PAHs containing up to four rings in the molecule. The significantly lower extraction efficiency of the *N*-doped CNT sorbent for high molecular weight PAHs is likely related to the very strong adsorption of the PAHs, which makes the desorption step very difficult. The interaction between the higher PAHs and the PDMS fiber surface is lower, and desorption is slightly easier. It was also observed that despite the high surface area, the activated carbon showed lower extraction efficiencies in comparison with the CNTs. Therefore, as discussed above, there were higher recoveries for high molecular PAHs using HMWCNTs; the reasons for this finding have not been clarified in the literature thus far. Thus, the existence of dissimilarity in the nature of bonding for low and higher molecular weight PAHs with MWCNTs was explored.

**Table 1:** Adsorption rate of PAHs onto different types of CNT (250 mL of deionized water sample, 0.5 μg L^−1^ of each PAH, 5% of *n*-propanol, 100 mg of CNT, contact time 60 min), RSD < 3%

PAHs	8MWCNT	50MWCNT	HMWCNT
Adsorption rate [%]			
Phenanthrene	99.4	100.0	100.0
Anthracene	100.0	100.0	99.1
Fluoranthene	100.0	99.4	100.0
Pyrene	99.5	100.0	98.9
Benzo(a)anthracene	99.7	99.3	100.0
Chrysene	100.0	100.0	100.0
Benzo(a)pyrene	100.0	98.9	100.0
Perylene	99.2	100.0	98.9
Indeno (1,2,3-cd) pyrene	100.0	100.0	100.0
Benzo[k]fluoranthene	99 5	100 0	100 0

### Similarity Analysis of PAH Molecules Based on QM Descriptors

Since the results described in the preceding sections seemed to indicate that the size of the PAH system is a key factor for predicting the recovery ability of the developed dSPE method, we decided to perform principal component analysis to investigate the similarities between the studied PAHs based on constitutional and QM descriptors calculated using the second-order Møller−Plesset (MP2) perturbational method and 6-311++G(d,p) basis set. The PCA model was calculated by using an autoscaled matrix of molecular descriptors (10 compounds × 14 descriptors; Table 2), The first two principal components explained 77% (PC1) and 11% (PC2) of the total variance in the data. Since the score plot of two PCs captures 88% of the overall variability in the data, it is expected to provide a reasonably accurate representation of the whole multi-dimensional space defined by the descriptors. As mentioned (in Section 2.5), the physical interpretation of a given PC can be assigned on the basis of contributions of the original descriptors to that PC (loading values).

**Table 2:** Molecular descriptors of 10 PAHs obtained based on the MP2/6-311++G(d,p) approach. The highest occupied molecular orbital energy (*E*_HOMO_) in electron volt; the lowest unoccupied molecular orbital energy (*E*_LUMO_) in electron volt; the energy difference between the LUMO and HOMO energy (GAP) in electron volt; mean atomic Sanderson electronegativity (scaled on Carbon atom) (Me); mean atomic polarizability (scaled on carbon atom) (Mp); number of carbon atoms (nC); number of atoms (nAT); number of hydrogen atoms (nH); percentage of hydrogen atoms (H%); number of rings (cyclomatic number), nCIC; number of circuits (nCIR); ring bridge count (*R*_brid_); ring fusion density (RFD); ring complexity index (RCI)

PAHs	*E*_HOMO_	*E*_LUMO_	GAP	Me	Mp	nAT	nH	nC	H%	nCIC	nCIR	*R*_brid_	RFD	RCI
Ant	− 7.008	1.045	8.053	0.976	0.742	24	10	14	41.7	3	6	2	0.286	1.286
B(a)A	− 7.122	1.011	8.133	0.977	0.752	30	12	18	40.0	4	10	3	0.333	1.333
B(k)F	− 7.178	1.000	8.178	0.978	0.768	32	12	20	37.5	5	18	5	0.500	1.450
B(a)P	− 6.728	1.004	7.732	0.978	0.768	32	12	20	37.5	5	22	6	0.600	1.500
Chr	− 7.385	1.014	8.399	0.977	0.752	30	12	18	40.0	4	10	3	0.333	1.333
Fluo	− 7.701	1.010	8.711	0.978	0.762	26	10	16	38.5	4	12	4	0.500	1.438
I	− 7.019	0.692	7.711	0.979	0.781	34	12	22	35.3	6	39	8	0.727	1.591
Per	− 6.580	0.753	7.333	0.978	0.768	32	12	20	37.5	5	22	6	0.600	1.500
Phen	− 7.723	1.023	8.745	0.976	0.742	24	10	14	41.7	3	6	2	0.286	1.286
Pyr	− 7.066	1.020	8.086	0.978	0.762	26	10	16	38.5	4	14	5	0.625	1.500

**Fig. 3:**
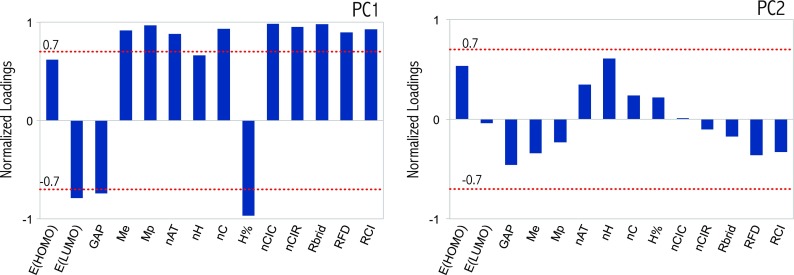
PCA loading values of the descriptors

According to the loading values (Fig. 3), PC1 represents the size of the PAHs. It is best seen in the case of ring descriptors, in which we observe a high correlation between PC1 and the ring count defining nCIC (number of rings) descriptor. In effect, PC1 divided the results into two groups, where the results for low molecular weight PAHs are located on the left side and those for PAHs containing at least five aromatic rings are located on the right side of the plot. The descriptors *R*_brid_ (ring bridge count), nCIR (number of circuits), RCI (ring fusion density), nAT (total number of atoms), and nC (total number of carbon atoms) are also closely related to the size of the particles. The descriptors Me and Mp describe respectively the cumulative electronegativity and polarizability of the atoms that constitute the compounds. PC1 values are higher for higher molecular weight PAHs that have higher polarizability and electronegativity. The last descriptors with a significant impact on the PC1 form are *E*_LUMO_ and GAP. The energy gap between HOMO and LUMO orbitals (GAP = *E*_LUMO_ − *E*_HOMO_) is an important indicator of kinetic stability. A large HOMO–LUMO gap implies high kinetic stability and low chemical reactivity because it is energetically unfavorable to add electrons to a high-lying LUMO or to extract electrons from a low-lying HOMO and so to form the activated complex of any potential reaction. Thus, PC1 will be higher for more reactive compounds (as the correlation coefficients of *E*_LUMO_ and GAP descriptors are negative). In contrast, PC2 is mostly related to the number of hydrogen atoms. Explicitly, it is readily apparent that PAHs with a high content of hydrogen atoms indicate higher values of PC2.

**Fig. 4:**
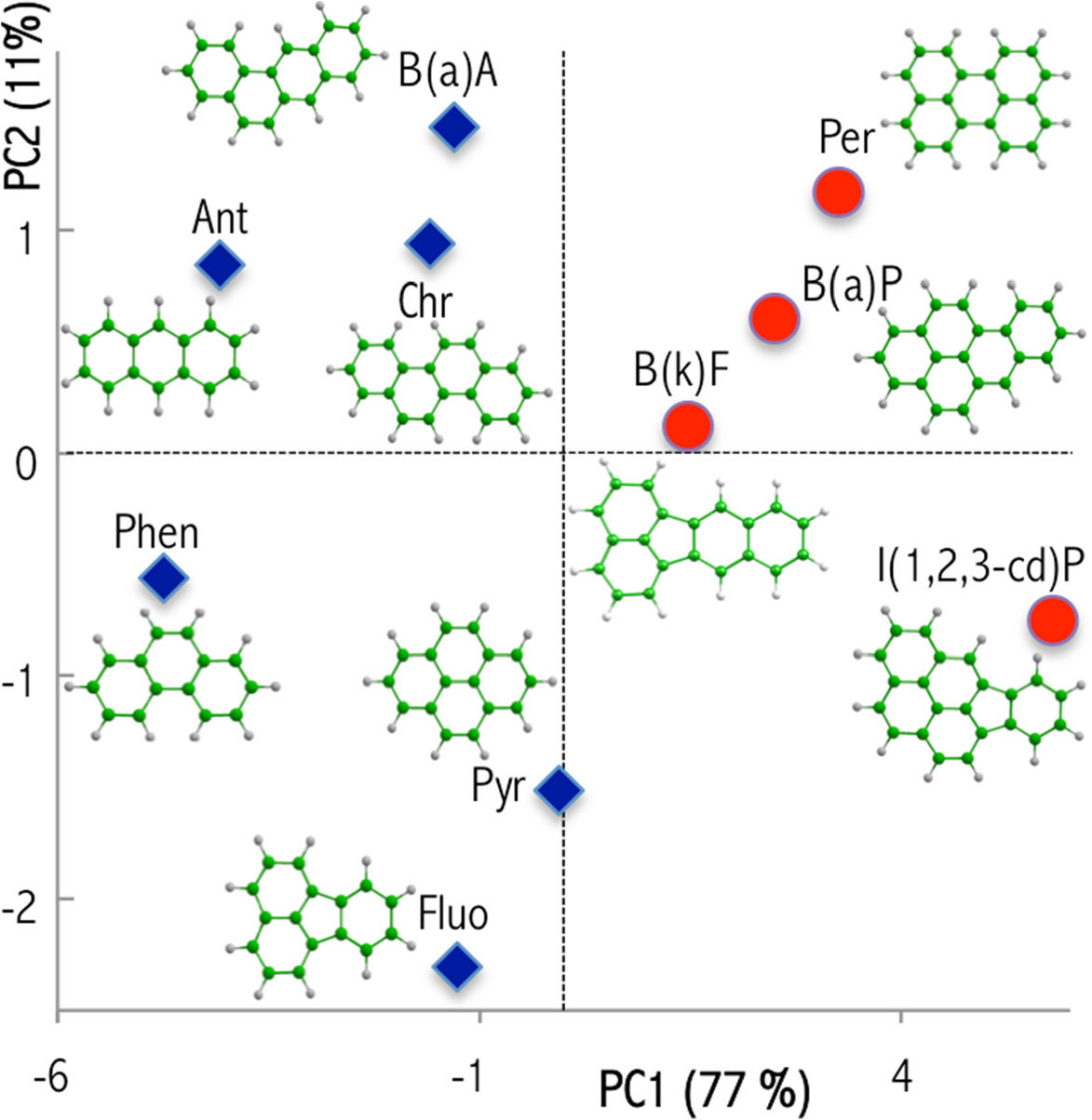
Score plot from two principal component analyses performed for 10 PAHs studied with the MP2/6-311++G(d,p) method. Shape codes: diamonds correspond to low molecular weight PAHs, and circles refer to PAHs containing at least five aromatic rings

**Fig. 5:**
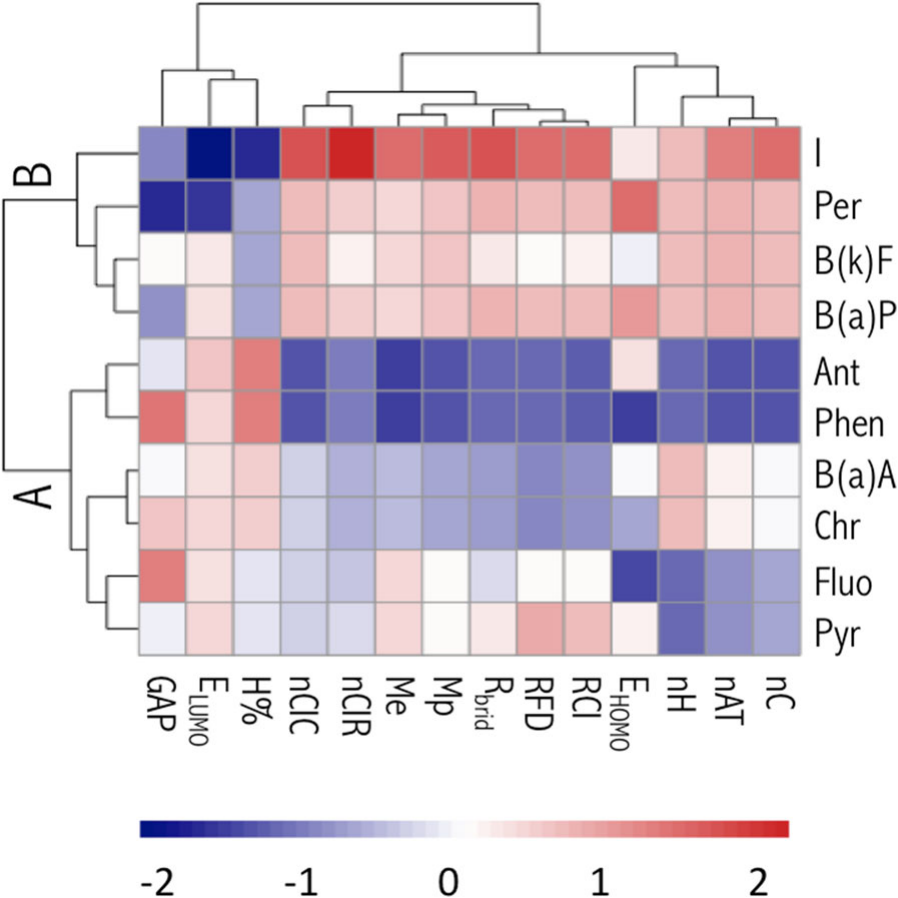
Hierarchical cluster analysis of 10 PAHs and the corresponding molecular descriptors

While analyzing the scatter plot (Fig. 4), it can be seen that within the space of molecular descriptors, the PAHs form distinct, separate clusters, which were also confirmed by hierarchical cluster analysis (HCA) results (Fig. 5). In the HCA variant that we have applied (supporting Section 2.2), compounds were grouped according to their pair correlations (compounds highly correlated with each other formed a particular cluster). Consequently, a clear distinction between PAH molecules is obtained by HCA (Fig. 5). Cluster A is comprised of low molecular weight PAHs, whereas cluster B consists of PAHs with at least five aromatic rings. Our observations are in good accordance with our interpretation of the meaning of the PC. These general conclusions are also a key to interpreting the results obtained with the developed dSPE method. All the remarks are helpful in understanding the final performance of the proposed dSPE methodology.

### Computational Modeling

In order to provide a better insight into the nature of interaction for PAHs and CNT surfaces, we decided to calculate the adsorption energy for PAH + CNT → PAH-CNT processes. We have arbitrarily chosen pyrene as the representative low molecular weight PAH system, whose adsorption energy of the corresponding adsorbed state (PAH-CNT) we compared with that for higher molecular weight PAHs (i.e., B(a)P, I(1,2,3-cd)P). Before adsorbing the PAHs on the different surfaces of CNTs, we calculated the structural parameters of pyrene (as a parent class of PAHs) in the gas phase and compared them with available experimental data to ensure that our calculations were accurate and reliable. The differences between *r*(*C*_i_ − *C*_j_)^PM6^ and the experimental value (Petersson et al. 1988) are in the 0.002–0.015 Å range, while the corresponding relative deviations (in %) calculated as |*r*(*C*_i_ − *C*_j_)^PM6^ − *r*(*C*_i_ − *C*_j_)^exp^| divided by *r*(*C*_i_ − *C*_j_)^exp^ indicate that the use of the PM6 method leads to errors of 0.14–0.98% (Table 3). Hence, we consider the level of theory employed in the present work as sufficient since the expected errors in the geometric structures of the species studied should not exceed 1%.

**Table 3:** The equilibrium bond distances in pyrene calculated using the PM6 method, the corresponding deviations (in Å) and relative deviations (in %) in bond distance (with respect to the experimental results; Petersson et al. 1988); for bond notation, see Fig. ESI.1

Parameters	PM6	Experiment	Dev (Å)	Dev (%)
r(C2-C3)	1.397	1.395	0.002	0.14
r(C3-C4)	1.404	1.406	− 0.002	0.14
r(C4-C5)	1.453	1.438	0.015	1.04
r(C5-C6)	1.355	1.367	− 0.012	0.88
r(C4-C15)	1.423	1.425	− 0.002	0.14
r(C15-C16)	1.444	1.430	0.014	0.98

### Structure and Thermodynamic Stability of PAH/Non-helical MWCNT Systems

The adsorption characteristics of three representative PAHs (i.e., Pyr, B(a)P, and I(1,2,3-cd)P) on three different carbon nanotubes: (i) zigzag-zigzag multi-walled CNTs (MWCNT(4,0)), (ii) chiral-chiral multi-walled CNTs (MWCNT(6,2)), and (iii) helical-CNTs (HCNT) were obtained by determining the preferred adsorption geometries and their corresponding adsorption energies, calculated from the relation below (Eq. 2):

2 $$ {E}_{\mathrm{ads}}={E}_{\mathrm{surf}/\mathrm{PAH}}-\left({E}_{\mathrm{surf}}+{E}_{\mathrm{PAH}}\right) $$

where *E*_suft/PAH_ is the total energy of the adsorbate–substrate system, *E*_surf_ is the energy of the clean surface of the CNT, and *E*_PAH_ is the energy of the isolated PAH molecule. The negative value for the adsorption energy means that the corresponding adsorbed state is thermodynamically more stable than the unbound state.

The adsorption parameters (binding energy (*E*_ad_), enthalpies (Δ*H*_r_^298^), entropies (Δ*S*_r_^298^), and Gibbs free energies (Δ*G*_r_^298^) of the adsorption processes) are summarized in Table 4, whereas the corresponding equilibrium adsorption geometries are shown in ESI (Figs. ESI.2 and ESI.3). Since the mutual parallel orientation of the subunits is always preferred while adsorbing PAHs to MWCNTs (as verified by examining the alternative vertical structures), one may observe such orientation with respect to each pair of PAH and MWCNT in PAH-MWCNT systems.

**Table 4:** Adsorption energies in gas phase (*E*_ads_ in kcal/mol) and in water solution (*E*_ads_^solv^ in kcal/mol) for the PAH adsorption to the carbon nanotube according to the PAH + CNT → PAH-CNT process. The results are obtained at the PM6 level

Adsorbate–substrate system	*E*_ads_	*E*_ads_^Solv^
Pyr-MWCNT(4,0)	− 45.59	− 39.45
B(a)P-MWCNT(4,0)	− 50.24	− 39.85
I(1,2,3-cd)P-MWCNT(4,0)	− 46.25	− 39.97
Pyr-MWCNT(6,2)	− 13.84	− 0.22
B(a)P-MWCNT(6,2)	− 59.17	− 43.32
I(1,2,3cd)P-MWCNT(6,2)	− 79.44	− 59.98
Pyr-HCNT	− 15.20	− 2.20
B(a)P-HCNT	− 9.85	− 7.75
I(1,2,3cd)P-HCNT	− 11.76	− 9.13

According to our findings, MWCNTs form stable adsorbate–substrate systems with the pyrene molecule, and the Pyr-MWCNT distance separating the two interacting moieties in the resulting equilibrium structure is equal to 3.354 Å for Pyr-MWCNT(4,0) and 3.500 Å for Pyr-MWCNT(6,2). The interaction of the pyrene molecule at the MWCNT(4,0) surface (*E*_ads_ = − 45.59 kcal/mol) as well as at the MWCNT(6,2) surface (*E*_ads_ = − 13.84 kcal/mol) does not involve a direct chemical bond formation with atoms of the MWCNT(4,0) or MWCNT(6,2) substrate, suggesting that van der Waals interactions (vdW) play an important role in stabilizing the adsorbed pyrene molecule. The small interaction energy for the Pyr-MWCNT(6,2) system seems to be consistent with the observation of low thermodynamic stability according to Pyr-MWCNT(6,2) → Pyr + MWCNT(6,2) process (Δ*G*_r_^298^ = 2.79 kcal/mol). The energetically most stable adsorption geometries for benzo(a)pyrene at MWCNTs are calculated to be parallel adsorption geometries with a total adsorption energy of − 50.24 and − 59.17 kcal/mol for MWCNT(4,0) and MWCNT(6,2), respectively. In the B(a)P-MWCNT adsorption geometry, the B(a)P system lies symmetrically around the interacting surface with the C atom at an almost equivalent C_PAH_–C_surf_ distance (3.333 and 3.429 Å), and the adsorbed B(a)P molecule, albeit slightly deformed, resembles the structure of the isolated B(a)P system. As we conclude, the adsorbed state composed of the MWCNT (i.e., MWCNT(4,0) or MWCNT(6,2)) and B(a)P is thermodynamically more stable than the unbound state as it possesses a negative value for the adsorption energy, and the Gibbs free energies of the reaction (Δ*G*_r_^298^) calculated for the B(a)P-MWCNT → B(a)P + MWCNT path processes are relatively large (35.19 kcal/mol for B(a)P-MWCNT(4,0) and 42.72 kcal/mol for B(a)P-MWCNT(6,2) kcal/mol; Table 5). Finally, we have identified slant adsorption geometries for the I(1,2,3-cd)P-MWCNT(6,2). In the slant adsorption geometry, the I(1,2,3-cd)P molecule lies at an angle of ~41° to the surface, normally interacting through two carbon–carbon π bonds (1.58 Å) with an adsorption energy of − 79.44 kcal/mol. A weaker adsorption energy, though negative (*E*_ads_ = − 46.25 kcal/mol), is predicted in the parallel I(1,2,3-cd)P-MWCNT(4,0) adsorption geometry where the I(1,2,3-cd)P molecule interacts with the surface carbon atoms through Van der Waals bonding (the closest C–C bond distance is calculated to be 3.407 Å). The I(1,2,3-cd)P tendency to interact with the MWCNT surface seems to be consistent with the observation of thermodynamic stability according to I(1,2,3-cd)P-MWCNT → I(1,2,3-cd)P + MWCNT processes (Δ*G*_r_^298^ read 35.18 and 55.66 kcal/mol at the MWCNT(4,0) and MWCNT(6,2) surfaces, respectively; Table 5).

**Table 5:** The enthalpies (Δ*H*_r_^298^, Δ*H*_r_^PCM^ in kcal/mol), the entropies (Δ*S*_r_^298^, Δ*S*_r_^PCM^ in cal/(molK), and the resulting Gibbs free energies (Δ*G*_r_^298^, Δ*G*_r_^PCM^ in kcal/mol) for the PAH-CNT → PAH + CNT processes (calculated for *T* = 298.15 K and *p* = 1013 hPa) for both gas and aqueous phases. The results are obtained at the PM6 level

Adsorbate–substrate system	Δ*H*_r_^298^	Δ*S*_r_^298^	Δ*G*_r_^298^	Δ*H*_r_^PCM^	Δ*S*_r_^PCM^	Δ*G*_r_^PCM^
Pyr-MWCNT(4,0)	42.44	20.43	36.35	37.32	29.73	28.45
B(a)P-MWCNT(4,0)	43.20	26.89	35.19	37.69	30.47	28.61
I(1,2,3-cd)P-MWCNT(4,0)	43.10	26.57	35.18	37.79	31.36	28.44
Pyr-MWCNT(6,2)	11.70	29.88	2.79	− 1.51	29.00	− 10.15
B(a)P-MWCNT(6,2)	55.38	42.46	42.72	38.71	37.53	27.53
I(1,2,3-cd)P-MWCNT(6,2)	73.13	58.60	55.66	53.64	58.15	36.30
Pyr-HCNT	6.51	30.62	− 2.62	− 2.17	34.01	− 12.21
B(a)P-HCNT	9.31	29.05	0.65	3.12	20.97	− 3.13
I(1,2,3-cd)P-HCNT	11.09	30.67	1.95	4.97	29.48	− 3.82

The different adsorption affinities exhibited between the high molecular weight PAHs (i.e., B(a)P and I(1,2,3-cd)P) and pyrene suggest that relatively strong π-electron-dependent polarizable interactions might have contributed to the adsorption of PAHs to MWCNTs. High molecular aromatic molecules might interact with the graphene surface more strongly due to their π-electron-rich property and flat conformation, while pyrene has weaker π-electron-coupling ability. Thus, the strong adsorption of B(a)P and I(1,2,3-cd)P compounds to MWNTs is attributed to the strong interaction between five aromatic rings of PAHs and the graphene sheets composing the surface of carbon nanotubes. Our implication is with accordance with earlier reports, which imply that electronic polarizability of the aromatic rings on the surface of CNTs might considerably enhance adsorption of the organic compounds to carbon nanotubes (Long and Yang 2001; Fagan et al. 2004; Chen et al. 2007).

### Comparison with Helical CNTs

Since the results described in the preceding sections seemed to indicate that the most important factor responsible for the adsorption process of the PAH molecules on carbon nanotubes is the ability of the CNT to bind with the PAH molecule, we decided to verify this observation by extending our theoretical studies to cover three additional systems (i.e., Pyr-HCNT, B(a)P-HCNT, and I(1,2,3-cd)P-HCNT) in which the MWCNT was replaced with a helical carbon nanotube (HCNT). The pyrene is found to adsorb only slightly weaker on the non-helical MWCNT(6,2) surface than on the helical carbon nanotube surface. Specifically, the vertical adsorption geometry gave a similar interaction strength, with the adsorption energy of − 15.20 kcal/mol for system involving HCNT, while that estimated for MWCNT(6,2) differs only by 1.36 kcal/mol (Table 4). On the other hand, the adsorption of benzo(a)pyrene or indeno(1,2,3-cd)pyrene to the helical CNT surface is predicted to be much weaker (in comparison to MWCNT), as indicated by the adsorption energies of the B(a)P + HCNT → B(a)P-HCNT (*E*_ads_ = − 9.85 kcal/mol) and I(1,2,3-cd)P + HCNT → I(1,2,3-cd)P-HCNT (*E*_ads_ = − 11.76 kcal/mol) processes. Hence, although these processes remain exothermic (due to the negative values of the adsorption energy), the predicted adsorption energies were found to be smaller (yet negative) in comparison to the corresponding PAH + MWCNT → PAH-MWCNT processes (PAH=B(a)P, I(1,2,3-cd)P). Furthermore, unlike on the non-helical MWCNT surface, the parallel adsorption geometry on the helical carbon nanotube surface does not involve direct chemical bond formation between the carbon atoms of the I(1,2,3-cd)P molecule and the CNT surface atoms, giving a weaker interaction with an adsorption energy of − 11.76 kcal/mol.

In summary, according to the PAH + CNT → PAH-CNT processes, all the adsorption processes considered were found to be energetically favorable (which is manifested by negative adsorption energy values). However, the adsorption energies (*E*_ads_) for PAHs and the helical carbon nanotube surface estimated for the B(a)P-HCNT and I(1,2,3-cd)P-HCNT are substantially less negative than those observed for PAH molecules interacting with the non-helical CNT. Namely, the *E*_ads_ calculated for the B(a)P-MWCNT(6,2) and I(1,2,3-cd)P-MWCNT(6,2) were respectively − 59.17 and − 79.44 kcal/mol, while values of only − 9.85 kcal/mol (B(a)P-HCNT) and − 11.76 kcal/mol (I(1,2,3-cd)P-HCNT) were found for the corresponding PAH-HCNT systems. Therefore, we conclude that the replacement of MWCNTs with HCNTs leads to PAH-HCNT systems in which the interaction energies are much smaller than those estimated for the corresponding PAH-MWCNT systems. This conclusion is additionally confirmed by low thermodynamic stability of I(1,2,3-cd)P-HCNT and B(a)P-HCNT systems toward PAH-HCNT → PAH + HCNT processes (the Δ*G*_r_^298^ read 0.65 and 1.95 kcal/mol for the I(1,2,3-cd)P-HCNT and B(a)P-HCNT, respectively; Table 5) and the Pyr-HCNT system instability due to negative value of the Gibbs free energy for the desorption process (Δ*G*_r_^298^ = − 2.62 kcal/mol for the Pyr-HCNT → Pyr + HCNT reaction; Table 5).

### Effect of Solvent on Adsorption Energy

Although the calculations carried out under the vacuum conditions (*ε* = 1) give a good measure of the raw interaction energies (Sikorska 2016), the geometric and energetic properties of adsorption processes may be somewhat different in a dielectric environment where *ε* ≠ 1. To estimate these effects, the polarizable continuum model (PCM) calculations (at the PM6 level of theory) were carried out in a simulated aqueous environment with fully optimized structures (Figs. ESI.5–7, supplementary material). The optimized geometries show that in the most cases, the PAH is oriented parallel to the surface of the CNT, indicating the existence of the π–π interaction. The adsorption energies for the PAH-CNT systems under study in aqueous solution (*E*_ads_^solv^ in Table 4) were found to be negative, which confirms that the PAH adsorption process onto the CNT surface is spontaneous. The variation trends presented by them are similar, both suggesting that the adsorption energies of the PAH-CNT systems are the highest in the gas phase and the solvent inclusion weakens the adsorption strength (by 6.1–19.5 kcal/mol; Table 4). The destabilization of the PAH-CNT systems in water solution is accompanied by the distance separating two interacting moieties (i.e., PAH and CNT) elongation by 0.136–0.353 Å; see Figs. ESI.2–7. The solvent effect does not change the stability order of the PAH-CNT systems. Hence, we observe a strong dependence between the carbon nanotube’s type and the value of adsorption energy calculated for the product involving this CNT. Namely, the lower *E*_ads_ values are always estimated for the PAH-CNT systems involving the non-helical carbon nanotubes. Thus, in the PAH-MWCNT(6,2)/PAH-HCNT pairs, the *E*_ads_^solv^ increases from − 43.32 to − 7.75 kcal/mol for B(a)P and the *E*_ads_^solv^ increases from − 59.98 to − 9.13 kcal/mol for I(1,2,3-cd)P adsorbates; see Table 4. The significant increase of the *E*_ads_^solv^ values caused by replacing the non-helical CNT with helical one confirms that the helical CNTs form weaker interaction with the higher molecular weight PAHs (such as B(a)P and I(1,2,3-cd)P) than the non-helical CNTs do. In case of the Pyr-MWCNT(6,2)/Pyr-HCNT pair, however, the *E*_ads_^solv^ values are similar and read − 0.22 and − 2.20 kcal/mol for Pyr-MWCNT(6,2) and Pyr-HCNT, respectively (Table 4).

Therefore, we assume that the PAHs bind more strongly to MWCNT in aqueous phase than HCNT do and their adsorption strength is directly correlated with the size (molecular weight) of the PAH counterpart. Increase in the molecular weight of the PAH molecule increases binding strength onto the non-helical CNT surface. In particular, for higher molecular weight (i.e., B(a)P and I(1,2,3-cd)P) compounds, the energies of adsorption are much lower in comparison with lower molecular PAH molecule (i.e., pyrene). Thus, molecular weight of the PAH plays a significant role and determines the strength of adsorption onto the non-helical MWCNT surface. In contrary, the adsorption energies for different PAHs are very similar when helical CNTs are used as a sorbent. Therefore, the adsorption of PAH by HCNT seems to be independent of the molecular weight of PAH.

In summary, the PCM results confirm both (i) the ability of carbon nanotubes to form stable adsorbate–substrate systems with PAHs in aqueous solution and (ii) the strong dependence of those systems stability on the type of carbon nanotube used. These conclusions are additionally confirmed by the Gibbs free energy (Δ*G*_r_^PCM^) values obtained for desorption processes (according to PAH-CNT → PAH + CNT reaction path) in the simulated aqueous environment. Specifically, the B(a)P-MWCNT and I(1,2,3-cd)P-MWCNT systems in aqueous solution were found to be thermodynamically stable and not susceptible to desorption process as the calculated Δ*G*_r_^PCM^ values span the 28.4–36.3 kcal/mol range (Table 5). From the other hand, Pyr-MWCNT(6,2) system was found to be thermodynamically unstable when desorption process is considered (the Δ*G*_r_^PCM^ for the Pyr-MWCNT(6,2) → Pyr + MWCNT(6,2) reaction reads − 10.15 kcal/mol; see Table 5). Because Δ*G*_r_^PCM^ > 0 enhances the stability of the PAH-CNT systems, our results confirm the strong interaction between higher molecular weight PAH molecules and the non-helical CNT in the aqueous phase. This phenomenon is not observed for lower molecular weight PAH (i.e., pyrene); instead, only locally stable adsorbate–substrate system is formed which is sustainable to desorption process. Furthermore, unlike on the non-helical MWCNT surface, both low and high molecular weight PAHs indicate weak binding to the helical carbon nanotube surface and the resulting PAH-HCNT systems are susceptible to separation processes (all calculated Gibbs free energies for desorption process (Δ*G*_r_^PCM^) were found to be negative; Table 5). Altogether, since the Gibbs free energy of desorption is directly related to the cost of regenerating the adsorbent, obtained Δ*G*_r_^PCM^ values strongly support the knowledge considering the performance of CNT materials for sensitive and selective analysis of PAHs in a water environment.

## Conclusions

In this paper, the possibility of using different types of carbon nanotube as the stationary phase in SPE was evaluated. For the first time, the usefulness of helical MWCNTs as the SPE sorbent for the isolation of PAHs was tested, and the high recovery values obtained using these nanotubes allow the conclusion that beyond the surface area, the shape of the fiber plays a significant role in the interaction with the analytes. In conclusion, an HMWCNT sorbent with GC–MS has a high analytical potential and allows the selective and sensitive analysis of PAHs at very low levels in a complex water environment. The method was demonstrated to be greatly applicable for the routine analysis of PAHs in surface waters. Moreover, the presented approach allows two PAH clusters to be distinguished according to their size. Explicitly, the structure of the PAHs with at least five rings significantly reduces the recovery ability in the case of MWCNTs. These compounds are the most hydrophobic among the hydrocarbons analyzed, and thus, they are the least water-soluble and show the highest tendency for sorption. The lower recovery values of the higher weight molecular PAHs are the effect of a strong bond with the non-helical CNTs.

## Electronic supplementary material

ESM 1 (DOCX 28141 kb)

## References

[CR1] Abdi H, Williams LJ (2010). Principal component analysis. Wiley Interdisciplinary Reviews: Computational Statistics.

[CR2] Abdolmohammad-Zadeh H, Sadeghi GH (2012). A nano-structured material for reliable speciation of chromium and manganese in drinking waters, surface waters and industrial wastewater effluents. Talanta.

[CR3] Al-Degs YS, Al-Ghouti MA, El-Sheikh AH (2009). Simultaneous determination of pesticides at trace levels in water using multiwalled carbon nanotubes as solid-phase extractant and multivariate calibration. J Hazard Mater.

[CR4] Bhadra M, Sae-Khow O, Mitra S (2012). Effect of carbon nanotube functionalization in micro-solid-phase extraction (μ-SPE) integrated into the needle of a syringe. Analytical and Bioanalytical Chemistry.

[CR5] Cai Y, Jiang G, Liu J, Zhou Q (2003). Multiwalled carbon nanotubes as a solid-phase extraction adsorbent for the determination of bisphenol A, 4-n-nonylphenol, and 4-tert-octylphenol. Analytical Chemistry.

[CR6] Chen W, Duan L, Zhu D (2007). Adsorption of polar and nonpolar organic chemicals to carbon nanotubes. Environmental Science & Technology.

[CR7] El-Sheikh AH, Insisi AA, Sweileh JA (2007). Effect of oxidation and dimensions of multi-walled carbon nanotubes on solid phase extraction and enrichment of some pesticides from environmental waters prior to their simultaneous determination by high performance liquid chromatography. Journal of Chromatography. A.

[CR8] El-Sheikh AH, Sweileh JA, Al-Degs YS (2008). Critical evaluation and comparison of enrichment efficiency of multi-walled carbon nanotubes, C18 silica and activated carbon towards some pesticides from environmental waters. Talanta.

[CR9] Fagan SB, Souza Filho AG, Lima JOG (2004). 1,2-Dichlorobenzene interacting with carbon nanotubes. Nano Letters.

[CR10] Fang GZ, He JX, Wang S (2006). Multiwalled carbon nanotubes as sorbent for on-line coupling of solid-phase extraction to high-performance liquid chromatography for simultaneous determination of 10 sulfonamides in eggs and pork. Journal of Chromatography. A.

[CR11] Fazelirad H, Ranjbar M, Taher MA, Sargazi G (2014). Preparation of magnetic multi-walled carbon nanotubes for an efficient adsorption and spectrophotometric determination of amoxicillin. Journal of Industrial and Engineering Chemistry.

[CR12] Fazelirad H, Ranjbar M, Taher MA, Sargazi G (2015). Preparation of magnetic multi-walled carbon nanotubes for an efficient adsorption and spectrophotometric determination of amoxicillin. Journal of Industrial and Engineering Chemistry.

[CR13] Frisch MJ, Trucks GW, Schlegel HB (2009). Gaussian 09.

[CR14] Gethard K, Mitra S (2011). Carbon nanotube enhanced membrane distillation for online preconcentration of trace pharmaceuticals in polar solvents. Analyst.

[CR15] Guan Y, Jiang C, Hu C, Jia L (2010). Preparation of multi-walled carbon nanotubes functionalized magnetic particles by sol-gel technology and its application in extraction of estrogens. Talanta.

[CR16] Hou X, Lei S, Qiu S (2014). A multi-residue method for the determination of pesticides in tea using multi-walled carbon nanotubes as a dispersive solid phase extraction absorbent. Food Chemistry.

[CR17] Hu C, He M, Chen B, Hu B (2015). Simultaneous determination of polar and apolar compounds in environmental samples by a polyaniline/hydroxyl multi-walled carbon nanotubes composite-coated stir bar sorptive extraction coupled with high performance liquid chromatography. Journal of Chromatography. A.

[CR18] Jakubus A, Paszkiewicz M, Stepnowski P (2017). Carbon nanotubes application in the extraction techniques of pesticides: a review. Critical Reviews in Analytical Chemistry.

[CR19] Long RQ, Yang RT (2001). Carbon nanotubes as superior sorbent for dioxin removal. Journal of the American Chemical Society.

[CR20] Ma J, Xiao R, Li J (2010). Determination of 16 polycyclic aromatic hydrocarbons in environmental water samples by solid-phase extraction using multi-walled carbon nanotubes as adsorbent coupled with gas chromatography-mass spectrometry. Journal of Chromatography. A.

[CR21] Masotti A, Caporali A (2013). Preparation of magnetic carbon nanotubes (Mag-CNTs) for biomedical and biotechnological applications. International Journal of Molecular Sciences.

[CR22] Menezes HC, de Barcelos SMR, Macedo DFD (2015). Magnetic N-doped carbon nanotubes: a versatile and efficient material for the determination of polycyclic aromatic hydrocarbons in environmental water samples. Analytica Chimica Acta.

[CR23] Miertus̃ S, Tomasi J (1982). Approximate evaluations of the electrostatic free energy and internal energy changes in solution processes. Chemical Physics.

[CR24] Miertuš S, Scrocco E, Tomasi J (1981). Electrostatic interaction of a solute with a continuum. A direct utilization of AB initio molecular potentials for the prevision of solvent effects. Chemical Physics.

[CR25] Mioduszewska K, Dołżonek J, Wyrzykowski D (2017). Overview of experimental and computational methods for the determination of the pKa values of 5-fluorouracil, cyclophosphamide, ifosfamide, imatinib and methotrexate. TrAC Trends in Analytical Chemistry.

[CR26] Nanotube Modeler Jc. (2015). Nanotube Modeler, JCrystalSoft, http://www.jcrystal.com/products/wincnt/.

[CR27] Niu H, Cai Y, Shi Y (2007). Evaluation of carbon nanotubes as a solid-phase extraction adsorbent for the extraction of cephalosporins antibiotics, sulfonamides and phenolic compounds from aqueous solution. Analytica Chimica Acta.

[CR28] Paszkiewicz M, Tyma M, Jakubus A, Stepnowski P (2016). Recent applications of carbon nanotubes as sorbents for the extraction of pharmaceutical residues. Current Analytical Chemistry.

[CR29] Paszkiewicz M, Caban M, Bielicka-Giełdoń A, Stepnowski P (2017). Optimization of a procedure for the simultaneous extraction of polycyclic aromatic hydrocarbons and metal ions by functionalized and non-functionalized carbon nanotubes as effective sorbents. Talanta.

[CR30] Petersson GA, Bennett A, Tensfeldt TG, et al (1988) A complete basis set model chemistry. I. The total energies of closed-shell atoms and hydrides of the first-row elements. 2193–2218.

[CR31] Popov VN (2004). Carbon nanotubes: properties and application. Materials Science & Engineering R: Reports.

[CR32] Praus P (2005). SVD-based principal component analysis of geochemical data. Open Chemical Engineering Journal.

[CR33] Pyrzynska K (2011). Carbon nanotubes as sorbents in the analysis of pesticides. Chemosphere.

[CR34] Pyrzynska K (2013). Use of nanomaterials in sample preparation. TrAC Trends in Analytical Chemistry.

[CR35] Ravelo-Pérez LM, Hernández-Borges J, Rodríguez-Delgado MA (2008). Multi-walled carbon nanotubes as efficient solid-phase extraction materials of organophosphorus pesticides from apple, grape, orange and pineapple fruit juices. Journal of Chromatography. A.

[CR36] Ravelo-Pérez LM, Herrera-Herrera AV, Hernández-Borges J, Rodríguez-Delgado MÁ (2010). Carbon nanotubes: solid-phase extraction. Journal of Chromatography. A.

[CR37] Scida K, Stege PW, Haby G (2011). Recent applications of carbon-based nanomaterials in analytical chemistry: critical review. Analytica Chimica Acta.

[CR38] Sikorska C (2016). When a nanoparticle meets a superhalogen: a case study with C 60 fullerene. Physical Chemistry Chemical Physics.

[CR39] Sikorska C, Puzyn T (2015). The performance of selected semi-empirical and DFT methods in studying C 60 fullerene derivatives. Nanotechnology.

[CR40] Suárez B, Santos B, Simonet BM (2007). Solid-phase extraction-capillary electrophoresis-mass spectrometry for the determination of tetracyclines residues in surface water by using carbon nanotubes as sorbent material. Journal of Chromatography. A.

[CR41] Suárez B, Simonet BM, Cárdenas S, Valcárcel M (2007). Determination of non-steroidal anti-inflammatory drugs in urine by combining an immobilized carboxylated carbon nanotubes minicolumn for solid-phase extraction with capillary electrophoresis-mass spectrometry. Journal of Chromatography. A.

[CR42] Sun N, Han Y, Yan H, Song Y (2014). A self-assembly pipette tip graphene solid-phase extraction coupled with liquid chromatography for the determination of three sulfonamides in environmental water. Analytica Chimica Acta.

[CR43] Trakakis G, Tasis D, Parthenios J (2013). Structural properties of chemically functionalized carbon nanotube thin films. Materials (Basel).

[CR44] Wang W-D, Huang Y-M, Shu W-Q, Cao J (2007). Multiwalled carbon nanotubes as adsorbents of solid-phase extraction for determination of polycyclic aromatic hydrocarbons in environmental waters coupled with high-performance liquid chromatography. Journal of Chromatography. A.

[CR45] Wepasnick KA, Smith BA, Bitter JL, Howard Fairbrother D (2010). Chemical and structural characterization of carbon nanotube surfaces. Analytical and Bioanalytical Chemistry.

[CR46] Wu H, Wang X, Liu B (2010). Flow injection solid-phase extraction using multi-walled carbon nanotubes packed micro-column for the determination of polycyclic aromatic hydrocarbons in water by gas chromatography-mass spectrometry. Journal of Chromatography. A.

[CR47] Zhao H, Wang L, Qiu Y (2007). Multiwalled carbon nanotubes as a solid-phase extraction adsorbent for the determination of three barbiturates in pork by ion trap gas chromatography-tandem mass spectrometry (GC/MS/MS) following microwave assisted derivatization. Analytica Chimica Acta.

