# Supplementary material for: Helical Multi-walled Carbon Nanotubes as an Efficient Material for the Dispersive Solid-Phase Extraction of Low and High Molecular Weight Polycyclic Aromatic Hydrocarbons from Water Samples: Theoretical Study
Source: Water Air Soil Pollut. 2018 Jul 14;229(8):253. doi: 10.1007/s11270-018-3884-0 (PMC6133110; doi:10.1007/s11270-018-3884-0)
Supplement: Supplementary file 1 — (DOCX 28141 kb) [file 11270_2018_3884_MOESM1_ESM.docx]

**Helical multi-walled carbon nanotubes as an efficient material for the dispersive solid-phase extraction of low and high molecular weight polycyclic aromatic hydrocarbons from water samples: theoretical study**

Monika Paszkiewicz^1,3^, Celina Sikorska^2,*^, Danuta Leszczyńska^3^, Piotr Stepnowski^1^

^1^ Department of Environmental Analytics, Protection, Faculty of Chemistry, University of Gdansk, Wita Stwosza 63, 80-308 Gdansk, Poland

^2^ Laboratory of Molecular Modeling, Department of Theoretical Chemistry, Faculty of Chemistry, University of Gdansk, Wita Stwosza 63, 80-308 Gdansk, Poland

^3^ Department of Civil and Environmental Engineering, Interdisciplinary Nanotoxicity Center, Jackson State University, 1400 John R. Lynch Street, Jackson, MS 39217, USA

*corresponding author: Celina Sikorska, celina.sikorska@ug.edu.pl, tel. +48 58 523 5351

**SUPPLEMENTARY INFORMATION**

1. **Materials and methods**

**1.1. Principle component analysis (PCA)**

Principal Component Analysis is a very popular mathematical technique, most commonly used to reduce the dimensionality of an analyzed dataset. Here, we present the general idea of PCA. In the initial data matrix, we can treat each feature as a separate vector, adding the same amount of information to the overall description of the samples. This amount of information is expressed as variance, and it is equal to 1 for each initial feature. By performing PCA, we can mathematically develop new, artificial features called Principal Components (PCs), which are eigenvectors of the covariance matrix, calculated from the original data matrix (Praus 2005; Abdi and Williams 2010). In this manner, we can treat PCs as compositions containing some fragments of all the initial features or, in the other words, a mixture of the initial features in different proportions. The first PC explains the largest possible amount of variance in the original data matrix, and each succeeding component accounts for as much of the remaining variability as possible. Thus, the percentage of total variance in the data explained by subsequent principal components decreases. In consequence, the total variance in the data is “compressed” in the first few principle components; the number of PCs is usually much lower than the number of original variables. Moreover, all PCs are orthogonal (uncorrelated with each other) by definition, which is very useful when similarities between the studied objects are studied (Praus 2005).

**1.2. Hierarchical cluster analysis (HCA)**

HCA is a grouping method, which allows arranging the tested objects into clusters, basing on the mathematically derived distances between them. Selecting appropriate distance measure and clustering technique define the exact purpose of analysis. In our work we performed HCA on PAHs represented on the linear maps, in order to provide some detailed information concerning their distribution. We used Euclidean distance and Ward’s clustering method.

**
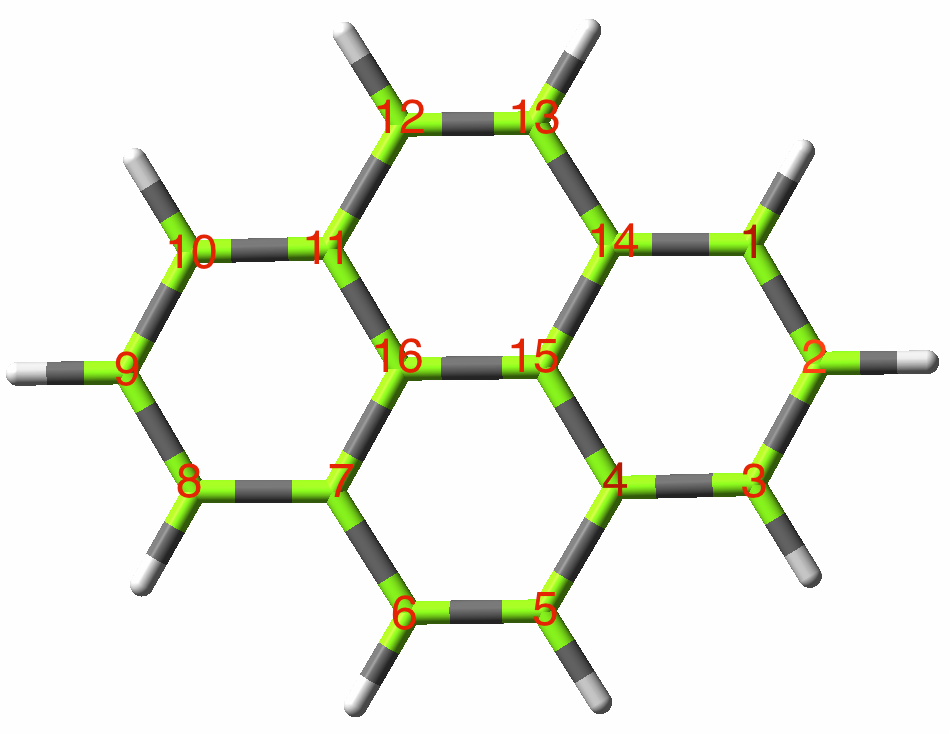
**

**Figure ESI.1**

**The equilibrium structure of pyrene calculated at the PM6 level of theory**

**Figure ESI.2.**

**Relaxed adsorption geometries of the (a) pyrene, (b) benzo(a)pyrene, and indeno(1,2,3-cd)pyrene on zigzag-zigzag MWCNT structure (tubes’ chirality indices: (4,0) and (10,0), tube-length=20 Å) calculated at the PM6 level. The side (left) and top (right) views are shown.**

**Figure ESI.3.**

**Relaxed adsorption geometries of the (a) pyrene, (b) benzo(a)pyrene, and indeno(1,2,3-cd)pyrene on chiral-chiral MWCNT structure (tubes’ chirality indices: (6,2) and (12,2), tube-length=20 Å) calculated at the PM6 level. The side (left) and top (right) views are shown.**

**Figure ESI.4.**

**Relaxed adsorption geometries of the (a) pyrene, (b) benzo(a)pyrene, and indeno(1,2,3-cd)pyrene on helical carbon nanotube (HCNT) structure (characterized by chiral indices (3,3)) calculated at the PM6 level. The side (left) and top (right) views are shown.**


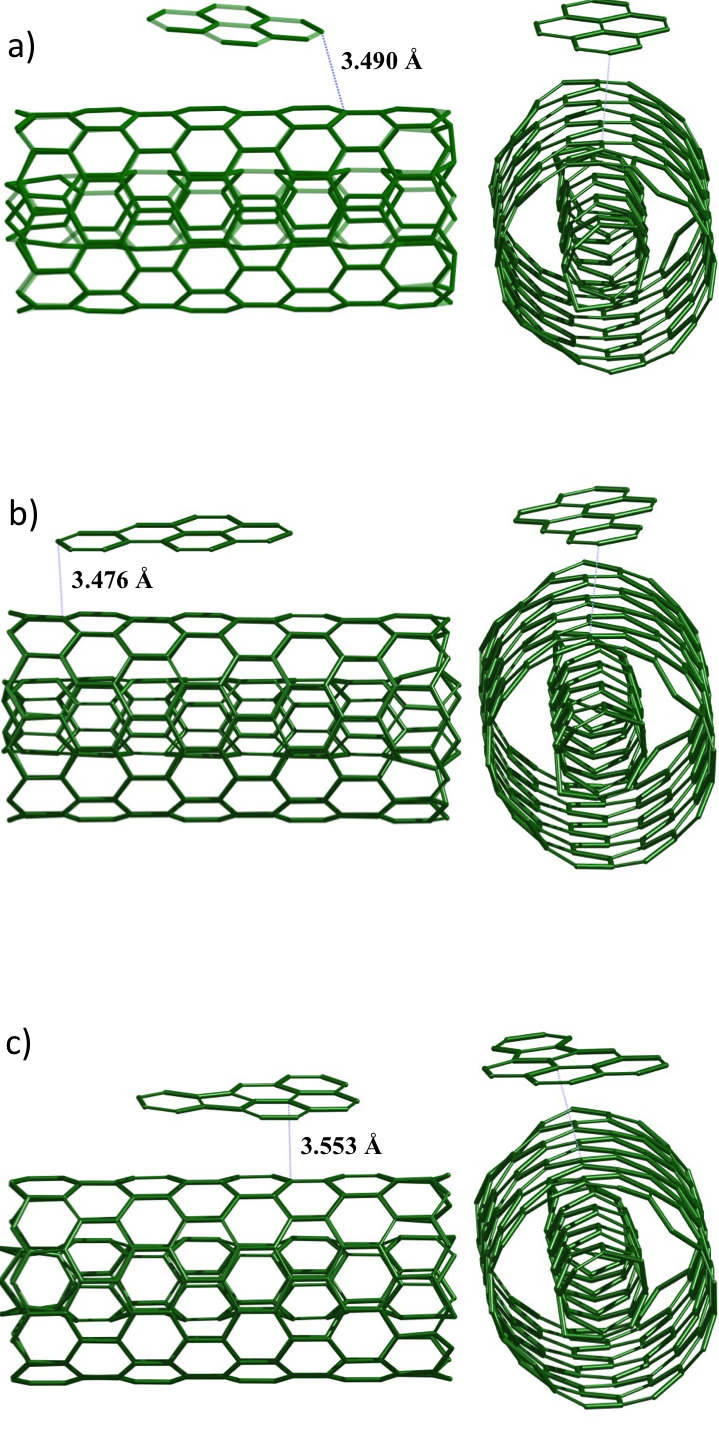


**Figure ESI.5. *Relaxed adsorption geometries of the (a) pyrene, (b) benzo(a)pyrene, and indeno(1,2,3-cd)pyrene on zigzag-zigzag MWCNT structure (tubes’ chirality indices: (4,0) and (10,0), tube-length=20 Å) calculated at the PM6 level within the polarizable continuum model (PCM) with water as solvent. The side (left) and top (right) views are shown.***


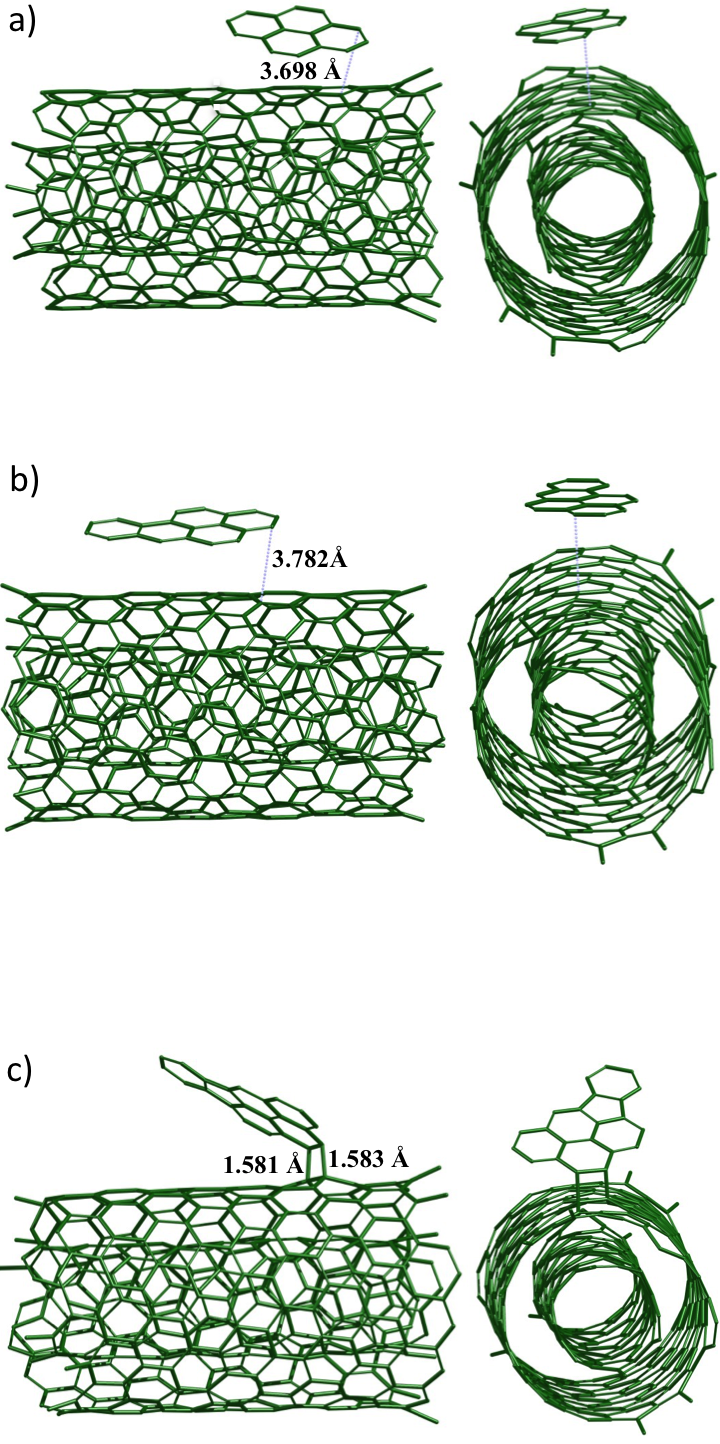


***Figure ESI.6. Relaxed adsorption geometries of the (a) pyrene, (b) benzo(a)pyrene, and indeno(1,2,3-cd)pyrene on chiral-chiral MWCNT structure (tubes’ chirality indices: (6,2) and (12,2), tube-length=20 Å) calculated at the PM6 level within the polarizable continuum model (PCM) with water as solvent. The side (left) and top (right) views are shown.***

***
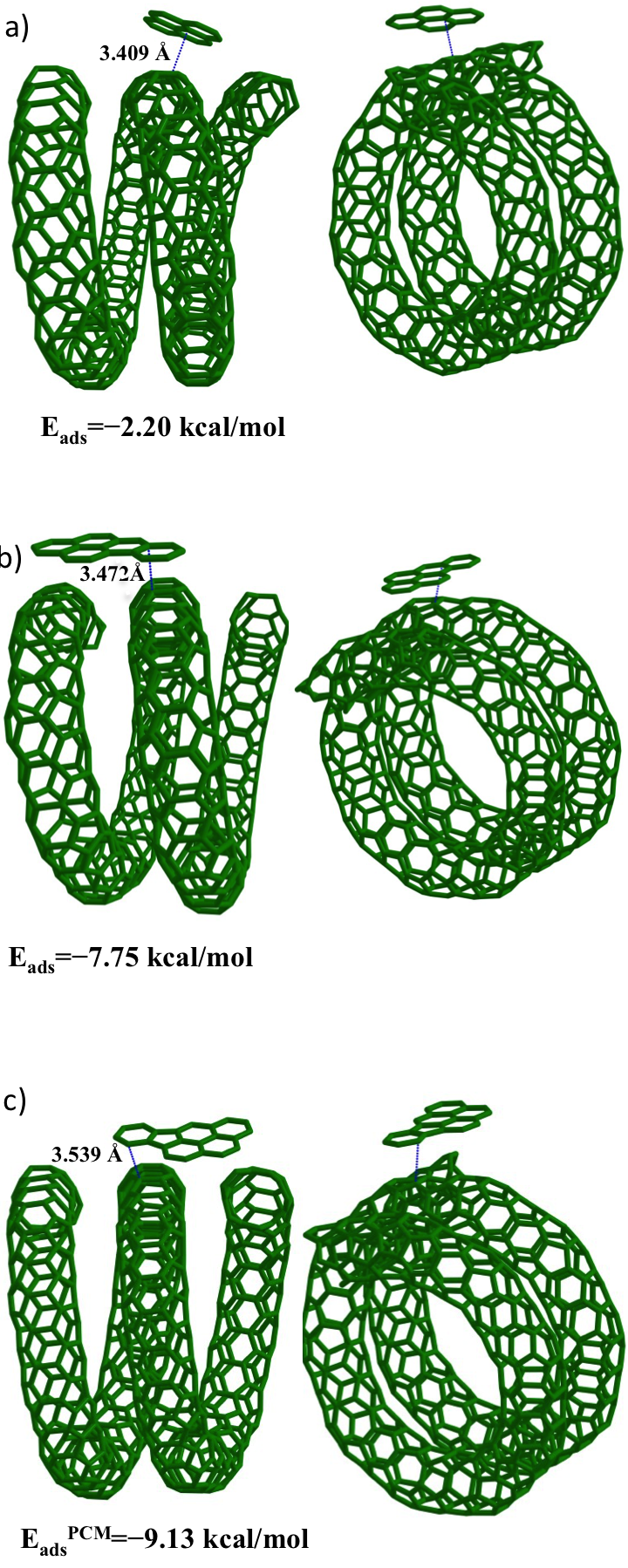
***

**Figure ESI.7. *Relaxed adsorption geometries of the (a) pyrene, (b) benzo(a)pyrene, and indeno(1,2,3-cd)pyrene on helical carbon nanotube (HCNT) structure (characterized by chiral indices (3,3)) calculated at the PM6 level within the polarizable continuum model (PCM) with water as solvent. The side (left) and top (right) views are shown.***

**References**

Abdi H, Williams LJ (2010) Principal component analysis. Wiley Interdiscip Rev Comput Stat 2:433–459 . doi: 10.1002/wics.101

Praus P (2005) SVD-based principal component analysis of geochemical data. Open Chem 3:731–732 . doi: 10.2478/BF02475200
